# CaMKII Is Involved in the Choline-Induced Downregulation of Acetylcholine Release in Mouse Motor Synapses

**Published:** 2017

**Authors:** A. E. Gaydukov, O. P. Balezina

**Affiliations:** Department of Human and Animal Physiology, Biological Faculty, Lomonosov Moscow State University, Leninskie Gory 1, bldg. 12, Moscow, 119234, Russia

**Keywords:** calcium-calmodulin-dependent protein kinase II, neuromuscular synapse, alpha7-nicotinic acetylcholine receptors, choline

## Abstract

We investigated the involvement of calcium-dependent enzymes, protein kinase C (PKC) and calcium-calmodulin-dependent protein kinase II (CaMKII), in the signaling pathway triggered by the activation of presynaptic alpha7-type nicotinic acetylcholine receptors by exogenous choline, leading to downregulation of the evoked acetylcholine (ACh) release in mouse motor synapses. Blockade of PKC with chelerythrine neither changed the evoked release of ACh by itself nor prevented the inhibitory effect of choline. The CaMKII blocker KN-62 did not affect synaptic activity but fully prevented the choline-induced downregulation of ACh release.

## INTRODUCTION

Choline comes from the products of the hydrolysis of acetylcholine (ACh) neurotransmitter by acetylcholinesterase in cholinergic synapses. Along with choline reuptake into the nerve terminals where it is recycled to synthesize ACh, choline plays an important role in the auto-regulation of ACh release by the feedback mechanism. This mechanism is associated with the ability of choline to selectively activate presynaptic alpha7-type nicotinic acetylcholine receptors (alpha7-nAChR) [1]. These receptors are abundant in central and peripheral synapses. Alpha7-nAChR permit the influx of sodium and calcium ions into the cell upon activation by choline and other agonists, leading to membrane depolarization, and also trigger diverse intracellular signaling cascades with the involvement of enzymes and channels [2]. In addition, it has been recently established that an alpha7-nAChR molecule contains an amino acid cluster that enables a functional interaction between alpha7-nAChR and G-proteins. This broadens the potential functions of alpha7-nAChR both as rapidly desensitizing ionotropic receptors and as special metabotropic receptors that trigger long-term signaling with long-term effects [3]. Therefore, these ambiguous consequences of presynaptic alpha7-nAChR activation in various types of synapses pose an important challenge that remains poorly studied. We have established recently that choline (0.1 mM) downregulates the evoked ACh release in mouse neuromuscular synapses via Ca^2+^-dependent Ca^2+^ efflux from the store through ryanodine receptors and the activation of the SK-type KCa channels of terminals, resulting in downregulation of ACh release [4]. Meanwhile, it was unclear whether Ca^2+^-dependent enzymes, such as protein kinase C (PKC) and/or calcium-calmodulin-dependent protein kinase type II (CaMKII), are involved in this cascade. Therefore, the aim of this study was to assess the changes in choline-evoked ACh release in mouse motor synapses, together with the blockade of calmodulin and Ca^2+^-dependent enzymes, protein kinase C (PKC), and calcium-calmodulin-dependent protein kinase II (CaMKII).

## MATERIALS AND METHODS

The experiments were conducted using isolated neuromuscular preparations from the diaphragm (*m. diaphragma – n. phrenicus*) of mature (P30) male mice of the 129/Sv strain provided by the Anokhin Institute of Normal Physiology, Russian Academy of Sciences (Moscow, Russia). A total of 16 mice were used. The mice were euthanized by quick decapitation. The mice were handled in accordance with Directive 86/609/EEC that regulates the use of laboratory animals. The procedure was approved by the Bioethics Commission of the Department of Biology, Moscow State University. All the experiments were conducted at room temperature of 20–22°C. Dissection of the neuromuscular preparation of the left half of the diaphragm with the phrenic nerve was performed according to the earlier described standard protocol [4]. Miniature endplate potentials (MEPPs) and multiquantal endplate potentials (EPPs) upon stimulation of the phrenic nerve were recorded using intracellular glass microelectrodes filled with 2.5 M KCl (the resistance at the microelectrode tip was 15–20 MΩ). First, MEPPs were recorded for 100 s, followed by recording of the EPPs in each synapse. The phrenic nerve was then stimulated with short trains of stimuli (50 stimuli 0.1 ms long each, frequency of 50 Hz). Signals were recorded using the Neuroprobe Amplifier Model 1600 (A-M Systems) and recorded using an L-Card E-154 analog-to-digital converter (with PowerGraph interface) into the PC hard drive. The data were then processed using the MiniAnalysis software (Synaptosoft). Controls included MEPP and EPP recordings from 5 or more different synapses; next, the substances under study were added to the perfusion solution in a particular order. The synaptic activity was registered during 1–1.5 h. At least 3 neuromuscular preparations were used in each experimental series. Choline, chelerythrine (Sigma, USA), W-7, KN-62 (Enzo Life Sciences, USA) were used. The amplitude, time parameters of MEPPs and EPPs, the MEPP frequency, and the quantal content of EPPs were estimated (the latter was calculated as the ratio between the mean EPP amplitude corrected for non-linear summation [5] and the mean MEPP amplitude). The statistical significance of the difference between the sample groups was assessed using the Student’s t-test and Mann–Whitney test. The significance level of the differences between two sample groups was 0.05 (*n* is the number of synapses studied).

## RESULTS AND DISCUSSION

Similar to our previous study [4], we used a 100-μM choline concentration to assess the presynaptic action of choline. This concentration is close to the choline concentration in the synaptic cleft during the hydrolysis of ACh and slightly exceeds the EC_50_ for activating alpha7-nAChR [6].

**Fig. 1:**
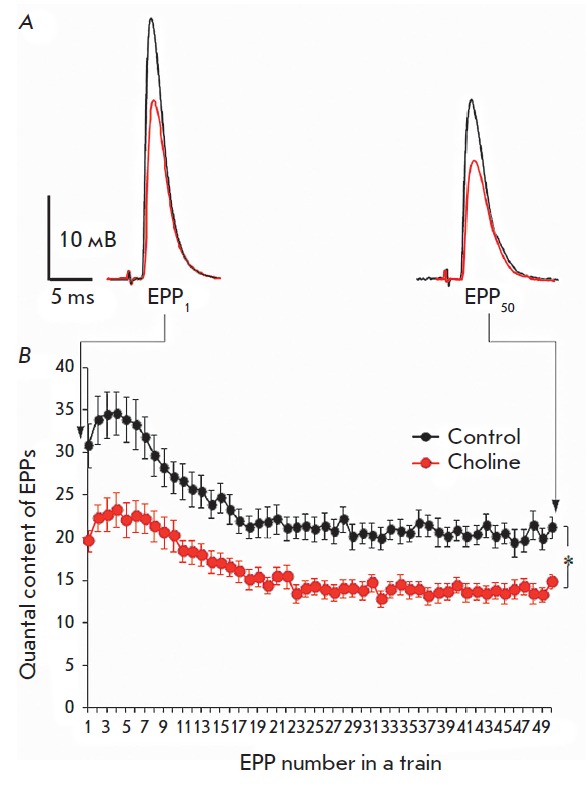
Downregulation of evoked ACh release by exogenous choline (100 μM) during rhythmic synaptic activity with a frequency of 50 Hz (1 s): *A *– the averaged recordings of the first (EPP1) and last (EPP_50_) EPPs in the trains in the control (black) and in the presence of choline (red). *B *– changes in the quantal content of EPPs during short-term rhythmic trains with a frequency of 50 Hz in the control and after the addition of choline (100 μM). The Y axis shows the quantal content of EPPs; the X axis shows the number of EPPs in the short train. * *p * < 0.05 with respect to the control values

Choline significantly changed neither the membrane potential of muscle fibers nor the spontaneous MEPP frequency. The mean amplitude of MEPPs in the presence of choline (1.08 ± 0.09 mV (*n *= 17)) also did not change significantly compared to that of the control (1.05 ± 0.08 mV (*n *= 15), *p *> 0.05). Short rhythmic stimulus trains (50 Hz, 1 s) led to characteristic changes in the amplitude and quantal content of EPPs in the train. The short-term facilitation of the synaptic transmission was followed by a depression in the form of a decreased amplitude of EPPs compared to the first EPP in the train, continuing into a lower stable level of EPPs (and the quantal content) compared to the first EPP (Fig*. *1). When pauses (at least 2 min long) were made between stimulus trains, the patterns of repeated trains were steadily reproduced in an individual synapse or other tested synapses. Application of choline reduced the EPP amplitude in the train because of the decay in the quantal content of EPPs. The quantal content of EPPs in the train in the presence of choline decreased significantly to 64–71% compared to the control (*p* < 0.05). In addition, the general pattern of the train remained unchanged (*Fig. 1*). The amplitude and the quantal content of EPPs decreased within 10–15 min after the administration of choline and remained at a lower level over the whole period during which choline was applied (for 45–60 min).

**The effects of PKC chelerythrine blocker**

**Fig. 2:**
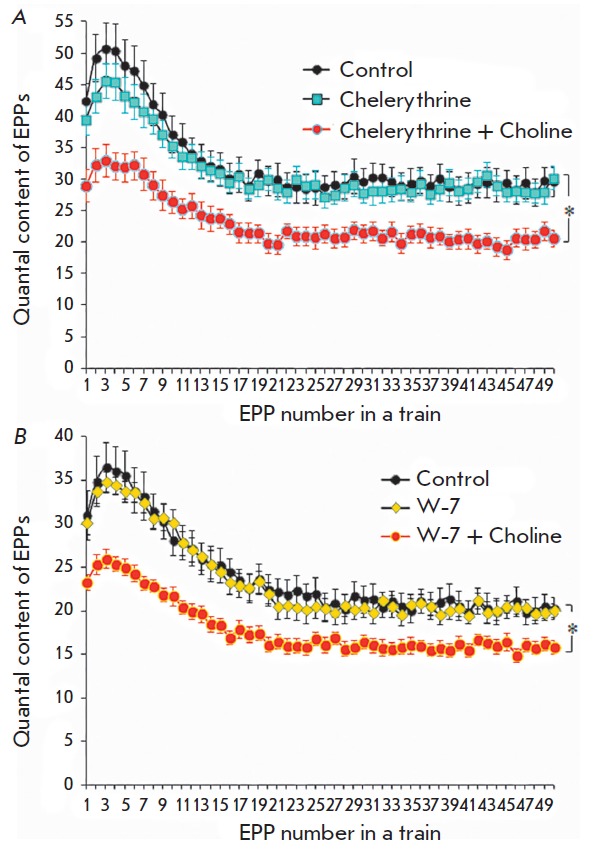
Changes in the quantal content during short-term rhythmic trains of EPPs with a frequency of 50 Hz: *A *– in the control, after chelerythrine (4 μM) was applied and after choline (100 μM) was added in the presence of preapplied chelerythrine. *B *– in the control (*n *= 17), after application of W-7 (10 μM) (*n *= 15) and when choline (100 μM) was added in the presence of pre-applied W-7 (*n *= 18). The Y axis shows the quantal content of EPPs; the X axis shows the EPP number in a short train. * *p * < 0.05 with respect to the control values

The application of PKC blocker chelerythrine on the muscle (4 μM) for 30–40 min did not significantly change the behavior of the bursting synaptic activity: neither the quantal content of EPPs in the train nor the train pattern (the initial facilitation, subsequent depression, and a plateau) changed significantly in the presence of chelerythrine (*p *> 0.05). In addition, chelerythrine had no impact on the inhibitory effects of choline in terms of the EPPs quantal content during bursting synaptic activity (*Fig. 2A*). Therefore, (1) the Ca^2+^-signals that enter the terminal upon choline-induced activation of alpha7-nAChR with subsequent release of the stored calcium and (2) the possible metabotropic signaling from alpha7-nAChR coupled with Gq-protein shown on other study objects [3] both do not activate PKC, and, thus PKC is not involved in the downregulation of ACh release in motor synapses. This agrees with our data and published citations showing that in motor terminals, PKC activation can be triggered by a calcium influx into the nerve endings via other Ca^2+^-channels, more specifically, via L-type Ca^2+^-channels, and also lead to the facilitation of ACh release [7].

**The effects of calmodulin blocker W-7**

Next, we studied the effects of choline subsequent to a preliminary inhibition of the regulatory activity of calmodulin using the W-7 calmodulin blocker (10 μM). The W-7 calmodulin blocker neither had a direct influence on synaptic transmission nor influenced significantly the downregulation effect of choline on the evoked ACh release. At the same time, choline-induced downregulation of ACh release in the presence of the calmodulin blocker was weaker than when only choline was added (*Fig. 2B*).

**Effects of CaMKII blocker KN-62**

**Fig. 3:**
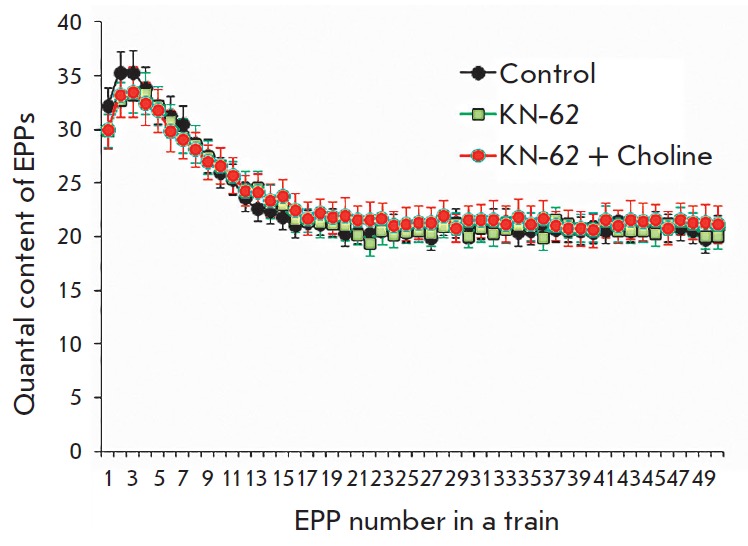
Changes in the quantal content during short-term rhythmic trains of EPPs with a frequency of 50 Hz in the control, after KN-62 (3 μM) was applied and when choline (100 μM) was added in the presence of pre-applied KN- 62. The Y axis shows the quantal content of EPPs, and the X axis shows the EPP number in a short train

In the final series of the experiments, we studied the possible activation and involvement of CaMKII in the inhibitory effects of choline. KN-62 (3 μM), a selective CaMKII blocker, was used. Neither statistically significant increments in the MEPP amplitude nor changes in the quantal content of EPPs in short trains were revealed during KN-62 solution perfusion of neuromuscular preparations for 30–40 min. Thus, the amplitude of MEPPs was 0.91 ± 0.05 mV (*n *= 20) in the control; it was 0.85 ± 0.04 mV (*n *= 23, *p *> 0.05) in the presence of KN-62 and was 0.83 ± 0.6 mV (*n *= 25) in the presence of choline and KN-62. However, in motor synapses when choline was added in the presence of preapplied KN-62, there was no significant decline in the amplitude and the quantal content of EPPs in a train compared to the control (*Fig. 3*).

We had previously revealed a choline-induced downregulation of ACh release triggered by the activation of presynaptic alpha7-nAChR, which suggests that activation of CaMKII can be involved in the downreg ulation of neurotransmitter release, along with other processes [4].

External calcium influx [8] and intracellular calcium efflux [9] have been shown to activate presynaptic CaMKII in the terminals of central and peripheral synapses, and possible downregulating and upregulating influences of CaMKII activation on transmitter and cotransmitter release have been revealed [9, 10]. A modulation of the evoked transmitter release occurs upon the activation of alpha7-nAChR by either endogenous or exogenous choline in CNS synapses. The generation of a calcium signal was recently described in response to the influence of choline on presynaptic alpha7-nAChR in hippocampal synapses; the calcium signals increased the amplitude of excitatory postsynaptic potentials, but the effects did not cause CaMKII activation and persisted in the presence of the KN-62 blocker [11]. We had first shown previously that in mouse peripheral synapses, the choline-induced activation of alpha7-nAChR downregulates the evoked ACh transmitter release and that this downregulation can be fully prevented by the blocking of ryanodine receptors or SK-channels [4]. This study is an important supplementation to these concepts. CaMKII was also found to participate in the auto-regulation of ACh release that occurs with the involvement of choline and alpha7-nAChR. With the revealed role of CaMKII in the auto-regulation of ACh release, we can add this kinase to the already described list of enzymes that play different roles in the signal transmission following alpha7-nAChR activation in different types of cells [3, 11]. Therefore, it is necessary to take into account the possibility of CaMKII activation when studying the role of alpha7-nAChR in the regulation of cellular processes.

We recently revealed CaMKII activation and its contribution to the enhancement of ACh release during calcium influx via L-type calcium channels, and this has been so far the only example of CaMKII involvement in the functions of neuromuscular synapses in rodents [12]. This study describes for the first time a fundamentally different way of CaMKII activation and participation in nerve end functions: i.e., the activation of alpha7-nAChR is associated with the involvement of CaMKII in downregulating ACh release. The role of CaMKII molecules residing close to alpha7-nAChR and intraterminal calcium stores can be to enhance and prolong the calcium signal, coupled with the function of ryanodine receptors, which is necessary for the activation of SK-type potassium channels.

Therefore, we have revealed for the first time a cascade of reactions in mouse motor nerve terminals that are triggered by a choline-induced activation of presynaptic alpha7-nAChR that downregulates ACh release. This cascade has been shown to rely on calcium release from stores, calcium-activated SK-type K+-channels, and the activity of the Ca^2+^-dependent enzyme CaMKII.

## Abbreviations

ACh: acetylcholine
alpha7-nAChR: nicotinic acetylcholine receptor of alpha7-type
MEPP: miniature endplate potential
EPP: endplate potential
PKC: protein kinase C
CaMKII: calcium-calmodulin-dependent protein kinase II
